# Pseudotumoral Lesion as a Manifestation of Autoimmune Pancreatitis

**DOI:** 10.7759/cureus.13931

**Published:** 2021-03-16

**Authors:** Catarina Parente, Rúben Reis, Daniela Rodrigues, António Cardoso, Joaquim Peixoto

**Affiliations:** 1 Internal Medicine Department, Centro Hospitalar Barreiro Montijo, Barreiro, PRT

**Keywords:** igg4 related pseudotumor, autoimmune pancreatitis (aip), diabetes, pancreatic neoplasm, igg4 disease

## Abstract

Autoimmune pancreatitis is a chronic and benign disease of autoimmune etiology that can occur isolated or constitute a manifestation of immunoglobulin G4 (IgG4)-related disease (types 2 and 1, respectively). It is a pathological condition that can mimic pancreatic cancer by presenting as a mass in imaging studies and provoking symptoms such as obstructive jaundice and dramatic weight-loss. The inflammatory infiltrates in the pancreas can also produce endocrine dysfunction leading to diabetes.

The authors report the case of a 68-year-old man that presented with unexplained weight loss and poorly controlled diabetes despite progressive pharmacological adjustments, with a later onset of obstructive jaundice, for which he underwent pancreaticoduodenectomy with the pre-operative diagnosis of pancreatic malignant neoplasm, which was posteriorly identified as type 1 autoimmune pancreatitis. In these cases, the differential diagnosis might be particularly challenging, requiring a high level of suspicion to avoid unnecessary procedures.

Corticosteroid therapy can lead to the resolution of symptoms as well as glycemic control, and it is the cornerstone of IgG4-related disease treatment. However, corticosteroid-sparing agents may be of interest to achieve clinical suppression.

## Introduction

Immunoglobulin G4-related disease (IgG4-RD) is a systemic immune-mediated condition characterized by inflammatory cell deposits in various organs leading to fibrosis and dysfunction, associated with increased serum IgG4 levels. Patients can present with subacute development of a mass in the affected organ, even though 60% to 90% of these patients have multiple organ involvement [1]. The prevalence of IgG4-RD is unknown since it is a relatively new entity and still largely underrecognized in clinical practice [2].

There are two types of autoimmune pancreatitis (AIP). The IgG4-RD associated pancreatitis is known as type 1 AIP. IgG4-related endocrinopathies are rare; however, in type 1 AIP, endocrine and exocrine dysfunction develop commonly, manifesting with hyperglycemia and steatorrhea [2]. AIP-related hyperglycemia may be completely controlled after glucocorticoid administration [3].

The authors report a case of autoimmune pancreatitis subjected to aggressive surgical treatment due to pseudotumoral presentation and uncontrolled diabetes resolved after corticosteroid therapy.

## Case presentation

**Figure 1:**
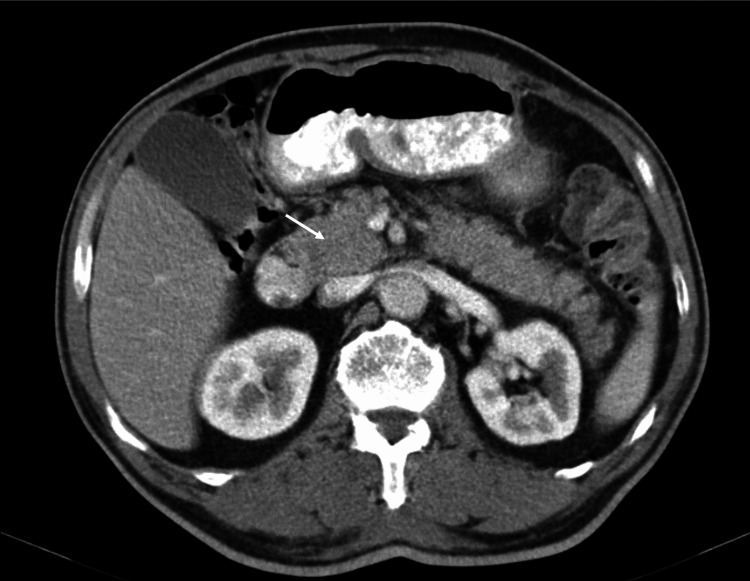
Abdominal CT scan The white arrow points towards a slightly spherical appearance of the cephalic pancreatic region.

A 68-year-old male with a medical history of diabetes mellitus under treatment with metformin presented with unintentional weight loss of 18 Kg over the last 10 months (6 Kg in the previous month), corresponding to 25.7% of his initial body weight. Laboratory evaluation revealed an increased erythrocyte sedimentation rate of 55 mm/h, glycated hemoglobin of 10.2%, and an elevated cancer antigen 19-9 (CA 19-9) of 50.8 U/mL. Even though the latter isn’t validated as a screening or diagnostic test, it was solicited amongst other parameters for clinical orientation. Liver and kidney function, as well as the other blood tests, were normal. His family practitioner decided to add gliclazide for metabolic control. The patient also underwent an upper gastrointestinal endoscopy and a colonoscopy that showed a non-bleeding angiodysplasia in the caecal top. A thoracic, abdominal, and pelvic computed tomography (CT) scan was obtained, which showed a roughly spherical appearance of the cephalic pancreatic region with 4.3 cm and 3.5 cm diameters (Figure 1). For further enlightenment, an abdominal magnetic resonance imaging (MRI) was executed that disclosed dilatation of intra- and extrahepatic bile ducts until the terminal portion of the common bile duct (CBD), which ended abruptly and eccentrically and a discrete area of hyposignal in T1 with 31 mm in the cephalic region of the pancreas.

A careful follow-up was conducted in the outpatient clinic, where an investigation was therefore being pursued. Despite these efforts, he was urgently admitted to the emergency department for obstructive jaundice with a total bilirubin level of 20.1 mg/dL and conjugated bilirubin of 14.2 mg/dL. A prompt endoscopic retrograde cholangiopancreatography (ERCP) was executed with alterations suggesting pancreatic neoplasm with biliary invasion. A plastic stent was placed in situ with good biliary drainage. The patient was then referred to a hepatobiliary surgical center, where he was admitted for a pancreaticoduodenectomy that was performed without reportable complications. The histology evaluation of the surgical specimen revealed pancreatic parenchyma with an inflammatory infiltrate intensely rich in plasmocytes that expressed IgG4 involving adipose tissue, duodenum, stomach, bile duct, and various reactive ganglia with the final histological diagnosis of autoimmune pancreatitis type 1. After the procedure, the plasmatic subclasses of IgG were measured without significant alterations, and the patient remained without specific treatment with good weight recuperation and resolution of symptoms.

However, despite the previously therapeutic adjustment of antidiabetic medication, metabolic control was not achieved with the need for progressive pharmacological adjustments, including insulin. A subsequent investigation revealed an IgG level of 1872 mg/dL (reference < 1822 mg/dL) and an IgG4 of 8530 mg/L (reference < 1400 mg/L). The patient was then started on corticosteroid therapy and methotrexate with an excellent clinical response.

## Discussion

There are a variety of pancreatic diseases that can form solid masses and mimic neoplasms. About 5%-10% of performed pancreatectomies with the preoperative clinical diagnosis of pancreatic carcinoma will be considered non-neoplastic lesions after anatomopathological examination (pseudotumors) [4]. Examples of such conditions include chronic pancreatitis, autoimmune pancreatitis, sarcoidosis, intrapancreatic accessory spleen, lymphoid hyperplasia, lipomatous pseudohypertrophy, lymphangioma, lymphoepithelial cyst, and endometriosis [4].

Autoimmune pancreatitis (AIP) is a nosological entity characterized by an autoimmune inflammatory process in which there is a prominent lymphocytic infiltrate leading to pancreas fibrosis and causing organ dysfunction. There are two types of AIP: type 1, which is a systemic disease associated with elevated serum IgG4 and tissue infiltration by IgG4+ plasma cells, and type 2, which is a specific pancreatic disorder not related to IgG4 [5]. These forms are clinically indistinguishable.

The clinical features of autoimmune pancreatitis are nonspecific and resemble other pancreatic diseases, including mimicking pancreatic cancer. Obstructive jaundice is the most common form of presentation [6]. AIP is frequently associated with exocrine and endocrine dysfunction, and diabetes mellitus was reported in 42%-78% of cases [7].

IgG4-related disease (IgG4-RD) is a systemic fibroinflammatory condition that affects multiple organs, mainly the pancreas, liver and bile ducts, salivary glands, kidneys, retroperitoneum, thyroid, aorta, and lymph nodes [8]. It is characterized by lymphoplasmacytic infiltrates rich in IgG4-positive plasma cells, and it occurs usually, but not always, with elevated IgG4 plasmatic levels [9]. About 10%-20% of patients have single organ involvement [10]. This inflammatory process responds to corticosteroid therapy, although fibrosis can lead to permanent organ damage.

## Conclusions

The present case report illustrates the evolution of an unknown IgG4-RD evolving to autoimmune pancreatitis that led to difficult metabolic control. The presentation as a pseudotumor highlights the need for clinical awareness of these conditions since invasive and potentially life-threatening procedures such as surgery should and could be avoided. Furthermore, the authors emphasize the use of methotrexate as a corticoid-sparing agent with a good clinical outcome.
